# Quantitative Edge Analysis of Pancreatic Margins in Patients with Chronic Pancreatitis: A Correlation with Exocrine Function

**DOI:** 10.3390/diagnostics13132272

**Published:** 2023-07-05

**Authors:** Maria Chiara Ambrosetti, Annamaria Grecchi, Alberto Ambrosetti, Antonio Amodio, Giancarlo Mansueto, Stefania Montemezzi, Giulia A. Zamboni

**Affiliations:** 1 Radiology Unit, Department of Pathology and Diagnostics, Azienda Ospedaliera Universitaria Integrata, 37126 Verona, Italy; mchiara.ambrosetti@gmail.com (M.C.A.); stefania.montemezzi@aovr.veneto.it (S.M.); 2 Institute of Radiology, Department of Diagnostics and Public Health, Policlinico GB Rossi, University of Verona, 37134 Verona, Italy; annamaria.grecchi88@gmail.com (A.G.); giancarlo.mansueto@univr.it (G.M.); 3 Department of Physics and Astronomy “Galileo Galilei”, University of Padova, 35131 Padova, Italy; alberto.ambrosetti@unipd.it; 4 Gastroenterology and Digestive Endoscopy Unit, The Pancreas Institute, Department of Medicine, G.B. Rossi University Hospital, 37134 Verona, Italy; antonio.amodio@aovr.veneto.it

**Keywords:** chronic pancreatitis, multidetector computed tomography, computer-assisted diagnosis

## Abstract

Background: Many efforts have been made to improve accuracy and sensitivity in diagnosing chronic pancreatitis (CP), obtaining quantitative assessments related to functional data. Our purpose was to correlate a computer-assisted analysis of pancreatic morphology, focusing on glandular margins, with exocrine function—measured by fecal elastase values—in chronic pancreatitis patients. Methods: We retrospectively reviewed chronic pancreatitis patients who underwent fecal elastase assessment and abdominal MRI in our institute within 1 year. We identified 123 patients divided into three groups based on the fecal elastase value: group A with fecal elastase > 200 μg/g; group B with fecal elastase between 100 and 200 μg/g; and group C with fecal elastase < 100 μg/g. Computer-assisted quantitative edge analysis of pancreatic margins was made on non-contrast-enhanced water-only Dixon T1-weighted images, obtaining the pancreatic margin score (PMS). PMS values were compared across groups using a Kruskal–Wallis test and the correlation between PMS and fecal elastase values was tested with the Spearman’s test. Results: A significant difference in PMS was observed between the three groups (*p* < 0.0001), with a significant correlation between PMS and elastase values (*r* = 0.6080). Conclusions: Quantitative edge analysis may stratify chronic pancreatitis patients according to the degree of exocrine insufficiency, potentially contributing to the morphological and functional staging of this pathology.

## 1. Introduction

Chronic pancreatitis (CP) is a complex, progressive, chronic inflammatory and fibrotic disease of the pancreas associated with structural—such as the gradual replacement of the acinar cells with fibrosis—and functional damage of the organ, which leads to abdominal pain, malabsorption, and weight loss—causing, in the long term, a reduction in quality of life, an increased risk of pancreatic ductal adenocarcinoma, and a reduction in life expectancy [1].

In Western countries, chronic pancreatitis typically affects males with a history of alcohol abuse and smoking—although it can have multiple etiologies and risk factors.

Its incidence has been growing—in part due to the improvement of diagnostic tools—and its prevalence is increasing thanks to advances in support therapies. In the United States, it has an annual incidence of five to eight cases per 100,000 adults and a prevalence of 42 to 73 cases per 100,000 adults [2]. The European data on its incidence and prevalence differ by geographical region. The reported global prevalence of chronic pancreatitis in Europe is between 16 and 163 per 100,000 adults [3,4]. The UK Biobank data show a prevalence of 163 per 100,000, a recent Danish study reports an incidence of 12.6 per 100,000 and a prevalence of 154 per 100,000, and an older French study reports an incidence of 7.7 per 100,000 and a prevalence of 26.4 per 100,000 adults.

In the later stages of chronic pancreatitis, pancreatic injury leads to progressive acinar replacement with fibrosis, and pancreatic atrophy is the final stage of the inflammatory process—with loss of lobulations and ductal abnormalities consisting of dilatations of the main pancreatic duct, with irregular margins and dilated side branches [5,6]. 

Clinically, it is characterized by chronic pain, exocrine insufficiency, and diabetes. 

Pancreatic exocrine insufficiency (PEI) is one of the major complications of chronic pancreatitis, with a prevalence that increases with disease duration; approximately half of patients will have developed exocrine insufficiency by 12 years after disease onset [7]. 

Functional non-invasive tests—such as fecal elastase tests—are used to investigate the pancreatic exocrine reserve to support diagnosis, stage patients, and follow up the course of treatment [8]. Pancreatic enzyme replacement therapy is recommended by several national societies when the clinical presentation is strongly suggestive of PEI to normalize digestion, alleviate PEI-related symptoms, and prevent malnutrition-related morbidity and mortality, as well as disease progression [9].

The probability of exocrine insufficiency in chronic pancreatitis can also be estimated based on pancreatic imaging findings in the absence of more advanced tests of pancreatic function [10,11]. 

The diagnosis of chronic pancreatitis is relatively easy in the late stage of the disease when irreversible damage to the gland has occurred, but can be especially challenging in the early phase, when the disease progress could theoretically be slowed or even halted by prompt clinical intervention and lifestyle changes.

Diagnosis is based on the evaluation of morphological changes in the pancreatic parenchyma and ductal system, identified by different imaging modalities—with MRI reported as being the most accurate for an early diagnosis [8]. The United European Gastroenterology evidence-based guidelines for the diagnosis and therapy of chronic pancreatitis define that, in the case of clinical suspicion of CP, the presence of typical imaging findings for CP with MRI/MRCP is sufficient for diagnosis [9]; The American College of Gastroenterology recommends cross-sectional imaging studies such as computerized tomography (CT) and Magnetic Resonance Imaging (MRI) with or without Magnetic Resonance Cholangiopancreatography (MRCP) as first-line diagnostic modalities.

In recent years efforts have been made, mainly with MRI, to improve the accuracy and sensitivity of the diagnosis of chronic pancreatitis—aiming in particular at a quantitative assessment that could overcome the traditional qualitative evaluation, be reproducible, and be related to functional data [12]. 

The first classification proposed for the quantitative assessment of the disease was the Cambridge classification, but it was originally developed for endoscopic retrograde cholangiopancreatography (ERCP) and subject to variable interpretation, with moderate interobserver agreement [13]. Its use has also been suggested for MRI and CT, but this is based only on ductal changes and does not consider parenchymal changes. Ductal imaging could not reflect the real level of parenchymal fibrosis or acinar cell loss, leading to delayed diagnosis of chronic pancreatitis in patients with only mild ductal abnormalities on MRCP. Thanks to technical advances in imaging techniques, it has become common to evaluate parenchymal and ductal changes together, with higher accuracy.

Secretin-stimulated MRCP (S-MRCP) allows one to obtain a morphological evaluation of the pancreatic gland and ducts, coupled with a quantitative functional dynamic assessment of the pancreatic exocrine volume reserve [14,15]. Although this gives the added value of a functional assessment, S-MRCP is not considered as standard in radiology practice as it increases the cost of the examination and prolongs the duration of the exam by a minimum of 15 min. 

DWI, MR relaxometry, T1-mapping, and ECV (extracellular volume fraction) are newer applications that may allow the exploration of a quantitative approach for the presence of fibrosis in patients affected by chronic pancreatitis. 

Diffusion weighted imaging, through the measurement of ADC values before and after secretin administration, has proven to be effective in differentiating healthy controls from patients with chronic pancreatitis, but shows weaknesses in grading patients in different stages of the disease [16,17,18]. 

Tirkes et al. found a significant difference between healthy controls and patients with mild chronic pancreatitis using T1-mapping, providing a valuable tool for early diagnosis—but its use for grading the disease was not investigated [19]. Similar results were obtained in another study that demonstrated the value of T1-mapping for staging all patients affected by chronic pancreatitis. The ECV fraction, since T1-shortening of the tissue reflects gadolinium-based contrast agent concentrations in the extracellular space, has been proposed for the quantification of intra- and extracellular components. Therefore, the decrease in the ratio between pancreatic acinar cells and fibrosis in the extracellular space could quantitatively assess the presence and severity of acinar cell loss and fibrosis in chronic pancreatitis, resulting in a diagnosis and the grading of the disease [20].

Pancreatic magnetic resonance elastography (MRE) has been demonstrated to be a promising technique for detecting pancreatic stiffness [21], and therefore for the assessment of fibrosis. Wang and colleagues have assessed the value of MRE and T1-mapping in diagnosing chronic pancreatitis, with MRE slightly outperforming T1-mapping [22]. Shi and colleagues have used MRE to predict the onset of post-operative fistulas after pancreatic surgery [23]. 

However, not all these applications are available in routine clinical practice. Moreover, there is no consensus on the use of these new methods applied to chronic pancreatitis.

Assessments of surface nodularity have been already proposed as an objective means of evaluating morphological changes occurring in liver disease, demonstrating a correlation with the fibrotic stage [24,25]. 

Evaluations of pancreatic margins have been demonstrated to correlate with the post-operative onset of fistulas after pancreaticoduodenectomy—a major complication after pancreatic resections, significantly increasing morbidity and mortality [26].

We hypothesized that the loss of glandular lobulations due to the inflammatory process in chronic pancreatitis could be quantitatively assessed by using this—thus introducing a simple, fast technique that does not require the acquisition of specific MR sequences or the administration of exogenous hormones or contrast agents. Therefore, the purpose of our study was to evaluate glandular changes with exocrine insufficiency, measured using fecal elastases.

## 2. Material and Methods

### 2.1. Patient Population

The database of all Patients with a clinical diagnosis of chronic pancreatitis who underwent fecal elastase (FE) tests between 1 January 2015 and 30 May 2020 at the Department of Gastroenterology of the University of Verona was investigated. The database included 2811 Patients. 

The inclusion criteria were:

- Patients with chronic pancreatitis from all etiologies, except for cancer-related obstructive pancreatitis;
- Patients who had undergone a fecal elastase test;
- Patients who underwent MRI at our Institute within a maximum of 1 year from the fecal elastase test, with images available for review on PACS.

The exclusion criteria were:

- Pediatric patients;
- No MR imaging available or imaging performed within more than 1 year from the fecal elastase test.

Informed consent for the utilization of clinical and radiologic data was provided by all patients, who were enrolled in a prospectively maintained registry (P-REGISTER Registry, n1271 CESC).

According to our laboratory and own experience, patients were divided into 3 groups based on their fecal elastase value:

- Group A, with fecal elastase > 200 μg/g (without exocrine pancreatic insufficiency);
- Group B, with fecal elastase between 100 and 200 μg/g (mild to moderate exocrine pancreatic insufficiency);
- Group C, with fecal elastase < 100 μg/g (severe exocrine pancreatic insufficiency).

### 2.2. Image Acquisition

MR examinations were performed on a 1.5 T scanner (Ingenia, Philips, Eindhoven, The Netherlands), with a combination of T1- and T2-weighted sequences, and dynamic acquisitions after weight-based Gadolinium-based contrast agent administration. We performed our analysis on the T1 FFE Dixon “water only” sequence (TR: shortest, TE: 1.79, slice thickness 5 mm, Voxel size 1.5 × 1.5 × 3 mm, FOV 400)—always included in the standard MRI protocol at our institute. 

### 2.3. Image Analysis

Two radiologists with 4 years and 14 years of experience in abdominal imaging, respectively, blinded to FE patient values, reviewed in consensus the MRI scans on diagnostic PACS workstations and selected from each scan one slice that included the majority of the parenchyma of the pancreatic body and tail, annotating the images for further reference. The body and tail were chosen for the analysis for two reasons: The first reason is that changes due to atrophy are typically seen earlier and better in this location. The second reason is that in these portions of the parenchyma, it is easier to have a free margin with an interface with fat—instead of having the parenchyma abutting other organs—while the ventral margin of the head is often in contact with the stomach or duodenum.

**Figure 1:**
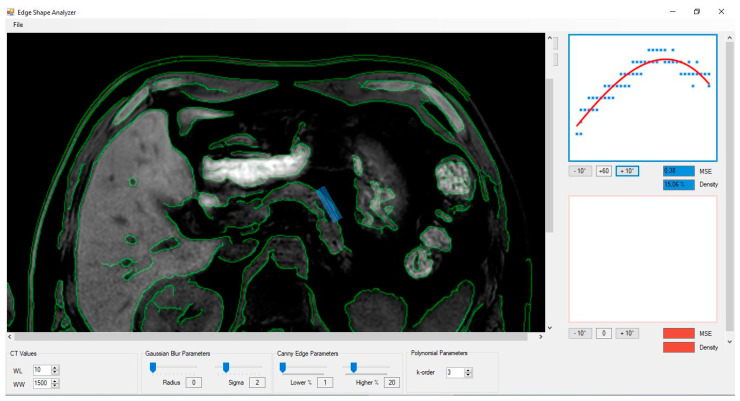
Screenshot of the graphical user interface of the used software, with a blue box drawn on the parenchymal surface. The graphic output of the results from the Canny edge detection (green margins) and the subsequent average boundary line (blue line in the box) are visible.

An in-house-developed software (Edge Shape Analyzer, v. 4.3) was used to perform a computer-assisted quantitative edge analysis. The digital non-enhanced images were processed with a Canny edge detector operator, which is a multi-stage image processing algorithm widely applied in various computer vision systems and also used in radiology applications [27]. Detectable pancreatic borders in the corresponding regions were extracted from digital non-enhanced images upon computation of the image color-intensity gradient, based on attenuation values measured on DICOM images pixel by pixel. When the gradient is higher than a certain threshold, the software marks that region as an organ border. The next step of the process is to draw a rectangular region of interest (ROI) on the pancreatic margins, obtaining the root mean square deviation SD of the actual border from the average boundary line, named the pancreatic margin score (PMS; Figure 1). The rectangular ROI was as big as possible in order to include the majority of the glandular margins.

Overall, low PMS values indicate mild fluctuations around the average boundary line, or, equivalently, a smooth edge. High PMS values instead provide a strong indication of edge roughness, with high fluctuations around the average boundary line.

Before analyzing in consensus the entire patient population, the two readers separately analyzed the first 20 cases to assess interobserver agreement.

### 2.4. Statistical Analysis

An ANOVA and Fisher’s exact-*t* test were used to compare the mean age and sex distribution between the three groups of patients.

Interobserver agreement was tested with the Cronbach Alpha test.

The PMS values obtained were compared across the three groups, using the Kruskal-Wallis test for continuous variables. The correlation between the PMS and fecal elastase values was tested with the Pearson test. A receiver operating characteristic (ROC) curve analysis was conducted to evaluate the accuracy of PMS in the differential diagnosis. *p* < 0.05 was chosen to indicate significance. All statistical analyses were performed with commercially available software (GraphPad Prism version 7.01 for Windows, GraphPad Software, La Jolla, CA, USA, www.graphpad.com, accessed on 16 February 2023).

## 3. Results

The final patient population included 123 patients (92 males and 31 females, mean age 55 ± 15 years). 

Preliminary interobserver agreement on the first 20 cases was very good (α = 0.89).

Group A included 41 patients (26 M, 15 F; mean age 50 ± 16 years). Group B included 40 patients (31 M, 9 F; mean age 55 ± 16 years). Group C included 42 patients (35 M, 7 F; mean age 59 ± 13 years). As expected, a significant difference in mean age was found between the three groups, with a higher mean age in group C (*p* = 0.03). No significant difference was found in the sex distribution in the three groups of patients.

Patient population characteristics are summarized in Table 1.

The causes of chronic pancreatitis in the patient population of this study were: CFTR and SPINK-1 mutation, non-cancer related obstructive pancreatitis, autoimmune pancreatitis, paraduodenal pancreatitis, hypertriglyceridemia, pancreas divisum morphology, and idiopathic. The distribution of etiologies for each group is summarized in Table 2.

### 3.1. PMS Values in Patients with Different Fecal Elastase Values

**Figure 2:**
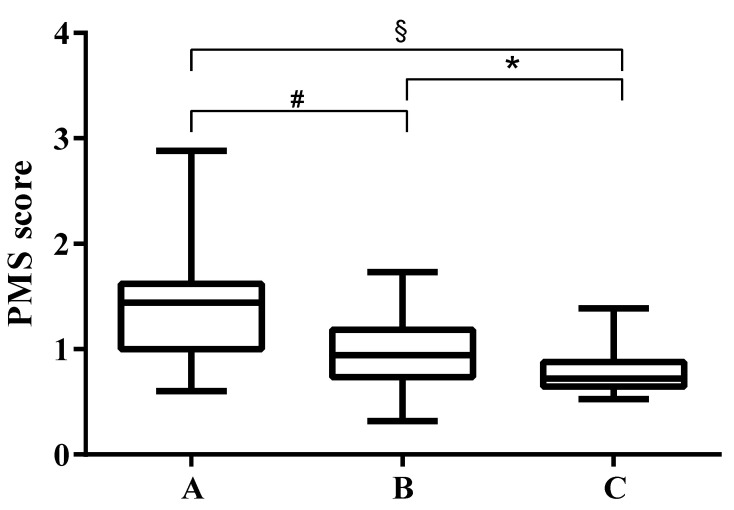
PMS (pancreatic margin score) values in the three groups of patients. A significant difference was found between values. * *p* = 0.01, # *p* = 0.0005, § *p* < 0.0001. Group A (without PEI), Group B (with mild to moderate PEI), Group C (with severe PEI).

**Figure 3:**
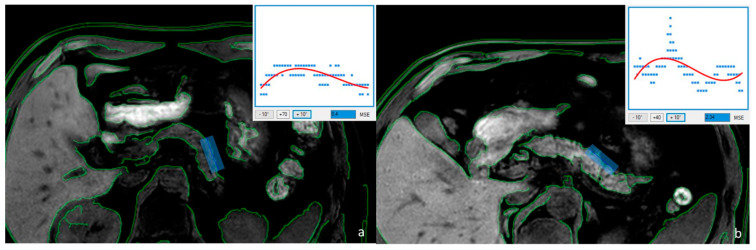
T1 FFE Dixon “water only” sequence of the abdomen, including the majority of the parenchyma of the pancreatic body and tail in patients with chronic pancreatitis with ((**a**); FE = 45 μg/g, male 63 years) and without ((**b**); FE = 320 μg/g, male 55 years) pancreatic exocrine insufficiency. PMS is calculated as the root of the MSE.

A significant difference in the pancreatic margin score values measured in the three groups of patients was observed: 1.37 ± 0.46 in group A, 0.96 ± 0.31 in group B, and 0.76 ± 0.17 in group C, respectively (*p* < 0.0001; Figure 2 and Figure 3). As expected, a lower elastase value (group C) was therefore associated with a smoother parenchymal margin, while a higher elastase value (group A) was associated with a higher roughness of the glandular edge.

**Figure 4:**
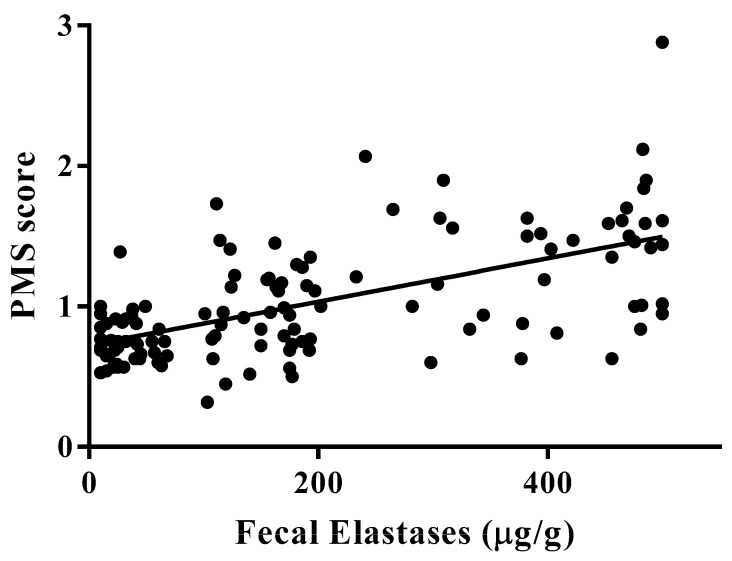
Positive correlation between pancreatic margin score and fecal elastase. *r* = 0.6080, *r*^2^ = 0.3697, *p <* 0.0001.

A significant positive correlation was observed between PMS scores and fecal elastase values (*r* = 0.6080, *r*^2^ = 0.3697; *p* < 0.0001; Figure 4).

### 3.2. ROC Analysis for Differential Diagnosis According to the Fecal Elastase Value

**Figure 5:**
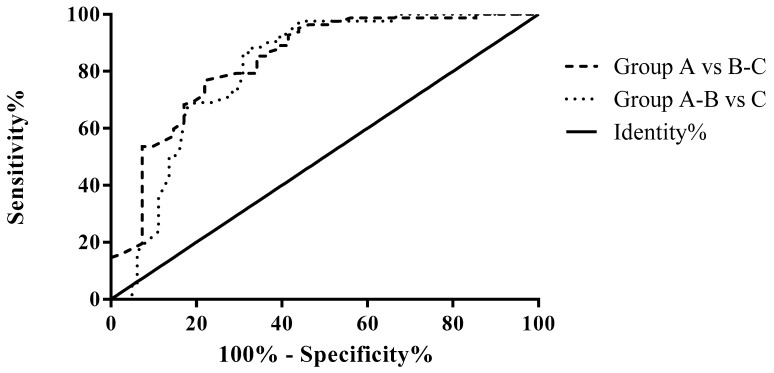
ROC curve of PMS values for differentiating patients in different groups based on fecal elastase values. Group A (without PEI), Group B (with mild to moderate PEI), Group C (with severe PEI).

The AUROC curve was 0.8373 for Group A vs. Groups B–C (i.e., patients with normal pancreatic exocrine function versus patients with pancreatic exocrine insufficiency; 95% CI 0.7575–0.9171; *p* < 0.0001). A cut-off PMS score of 1.4 provided 95.12% sensitivity and 56.10% specificity in differentiating Group A vs. Groups B–C, i.e., identifying patients with normal pancreatic exocrine function (Figure 5, Table 3).

The AUROC curve was 0.8094 for Groups A–B vs. C (i.e., patients without or patients with mild pancreatic exocrine insufficiency versus patients with severe pancreatic exocrine insufficiency; 95% CI 0.7338–0.8850; *p* < 0.0001). A cut-off PMS score of 1.005 provided 97.62% sensitivity and 55.56% specificity in identifying patients with severe exocrine insufficiency (Figure 5, Table 3).

**Figure 6:**
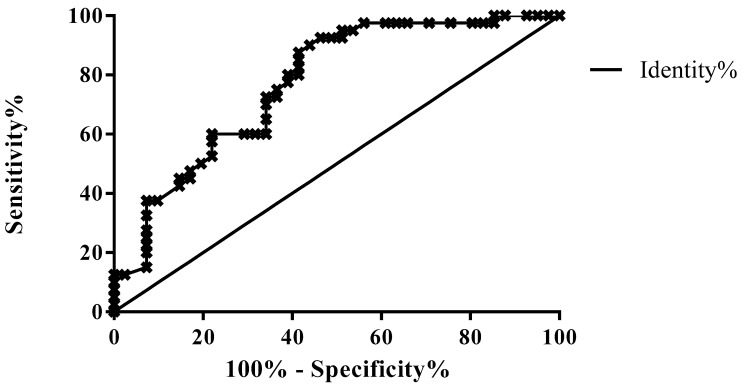
ROC curve of PMS values for differentiation between patients with normal pancreatic function (group A) and with mild pancreatic exocrine insufficiency (group B).

When comparing only patients without (group A) or with mild (group B) pancreatic exocrine insufficiency, the AUROC curve was 0.7701 (95% CI 0.6675–0.8727; *p* < 0.0001); a cut-off PMS score of 1.205 had 80% sensitivity and 64.98% specificity in differentiating between these two groups of patients (Figure 6, Table 3).

## 4. Discussion

Chronic pancreatitis is a progressive inflammatory disease associated with structural and functional damage to the pancreas, which in the later stages is associated with scarring and remodeling of the parenchymal edge [1], when acinar glands are replaced with fibrosis and pancreatic atrophy occurs. During imaging, this appears as a loss of lobulations coupled with ductal abnormalities, consisting of dilatations of the main pancreatic duct with irregular margins and dilated side branches. 

One of the major complications of chronic pancreatitis is pancreatic exocrine insufficiency, the prevalence of which increases with disease duration. Among the available functional non-invasive tests, the fecal elastase test is currently used to investigate pancreatic exocrine reserve to support diagnoses, stage patients, and follow up the course of treatment.

An accurate diagnosis in the early stage of chronic pancreatitis, when the pathological process can be stopped by prompt clinical intervention and lifestyle changes, remains difficult in the absence of visible structural changes in the parenchyma [12].

In recent years, many efforts have been made to obtain a more accurate assessment of morphological changes, even in the earlier stages of the disease—with computed tomography, magnetic resonance imaging, magnetic resonance cholangiopancreatography, endoscopic ultrasonography, and ultrasound recognized as valid imaging techniques. In particular, MRI has been used to obtain a quantification of the damage that occurs in the disease, which could have added value over traditional qualitative evaluations.

Tirkes et al. have demonstrated the usefulness of the extracellular volume fraction (EVF) and T1-mapping as biomarkers that correlate with the degree of pancreatic fibrosis and glandular functional reserve [28]. The same authors also assessed the value of T1-mapping in diagnosing mild chronic pancreatitis—concluding that T1-mapping seems to be a promising quantitative method for identifying fibrosis in the early stages of chronic pancreatitis [19,20]. Despite its reliability, T1-mapping is not a commonly available sequence; therefore, it is not easily incorporated into clinical practice. 

The use of DWI and ADC values—in particular the time to peak of ADC values after secretin stimulation—has proven to be useful as a functional quantitative parameter for detecting and staging glandular insufficiency [16,17,18]. Akisik et al. reported higher mean non-enhanced and maximum secretin-enhanced ADC values in healthy controls than in patients with mild or severe chronic pancreatitis; a significant difference, however, was not observed between patients with mild and severe CP. In patients with chronic pancreatitis, pancreatic ADC values may be useful in the diagnosis and assessment of the severity of the disease, while the ADC response to secretin administration has not been demonstrated to be useful [18].

In routine practice, the evaluation of the functional pancreatic reserve with MRCP after secretin stimulation has been used for years as a semi-quantitative method—assessing the changes in the diameter of the main pancreatic duct and the output of pancreatic juices in the duodenum [29]. This analysis was, however, focused only on pancreatic ductal changes and not on glandular parenchymal evaluation.

In both cases, the high cost of the secretin and the addition of a supplementary sequence with prolonged scan times were the downsides of these techniques. 

In contrast to these approaches, PMS can be measured without acquiring specific sequences and can even be applied to a retrospective analysis—allowing a comparison of serial values in the same patient to assess the evolution of the disease over time.

The idea of performing a quantitative analysis of anatomical margins was first proposed by Smith et al. with the creation of the Liver Nodularity Score (LNS). LNS is a computer-based quantitative method for analyzing hepatic margins on routine CT images, focused on detecting the presence of cirrhotic nodules [24,25]. It has proven to be an effective, rapid, and non-invasive tool for the diagnosis and staging of hepatic fibrosis and for predicting future hepatic decompensation and death. One of its main advantages is the retrospective use of previous CT images, allowing an evaluation of any intervening changes. 

With the same purpose, we wanted to elaborate a score for pancreatic margins with a quantitative approach to study the fibrosis and degeneration of the pancreatic parenchyma, and its correlation with the main test used to assess exocrine insufficiency—which is the fecal elastase measure. We applied it to MRI instead of CT because MR, according to guidelines, is the best modality for the early diagnosis of chronic pancreatitis [8]. We chose a T1-WI FFE Dixon “water only” sequence for its anatomical detail, because in this sequence, the glandular margins are best offset by the strong hypointensity of the fat surrounding the pancreas; the software, however, will also function on other MR sequences, and on CT images.

The assessment of surface nodularity has been already proposed as an objective means of evaluating morphological changes of the pancreatic parenchyma, demonstrating a correlation with the onset of post-operative fistulas in pancreatic surgery, which is also correlated with fecal elastase values [26,30]. Higher PMS values, indicative of higher glandular edge coarseness, are associated with a higher probability of post-operative fistula onset after pancreatic surgery. At the same time, higher fecal elastase values are correlated with a higher incidence of postoperative pancreatic fistulas.

In this retrospective study, we used our in-house-developed software to analyze the pancreatic margins to obtain a computer-based quantitative measurement of the loss of lobulations during routine MR imaging of patients with chronic pancreatitis and to stratify patients according to the severity of the disease.

A significant difference between PMS values was found in the three groups of patients, divided based on fecal elastase values. The highest values were obtained in patients with a normal value of fecal elastase, and this could be related to the greater coarseness of the margins—indicating that lobulations were preserved, as in the normal pancreas and in the early stage of chronic pancreatitis. On the contrary, lower values were seen in patients with severe pancreatic insufficiency, where the actual boundary line is more similar in shape to the central line of the ROI, revealing a loss of physiological pancreatic lobulations. The PMS could, therefore, be able to reflect one of the major architectural changes occurring during the development of chronic pancreatitis, i.e., the replacement of acinar parenchyma with fibrosis.

We observed a positive correlation between PMS values and fecal elastase—a well-established biomarker in the evaluation of chronic pancreatitis. The pancreatic margin score could, therefore, be used to investigate pancreatic function. Moreover, unlike fecal elastase, which can give false positive results in patients suffering from malabsorption from non-pancreas-related causes, the pancreatic margin score could be specifically associated with chronic pancreatitis.

We identified a PMS cut-off value (1.4) that enabled us to identify patients with pancreatic exocrine insufficiency (FE < 200 μg/g). Of even more relevance, a PMS cut-off value was also found for the identification of patients with only mild pancreatic exocrine insufficiency (with FE between 100 and 200 μg/g), in which, appropriate therapeutic strategies could slow down or stop the disease progression. This could possibly impact the prognosis of patients affected by chronic pancreatitis in the early stages, and should be further investigated prospectively.

Some limitations to our study must be acknowledged: Mainly, the study included a relatively small patient population from a single institution, leading to intrinsic limitations (participation bias, image-based selection bias); therefore, additional multicenter prospective studies are warranted to confirm these preliminary findings. 

## 5. Conclusions

In our single-center series, quantitative edge analysis with dedicated software appears to be able to stratify patients with chronic pancreatitis according to the degree of exocrine insufficiency defined by a measure of fecal elastase. This could potentially contribute to the morphological and functional staging of this disease, with the future goal of being able to make an early diagnosis. Further studies are warranted to assess reproducibility in multicenter cohorts, with patients studied using different MR scanners.

## Figures and Tables

**Table 1:** Patient population characteristics. PEI (pancreatic exocrine insufficiency).

	Group A (Without PEI)	Group B (With Mild to Moderate PEI)	Group C (With Severe PEI)
TOT	41	40	42
M	26	31	35
F	15	9	7
Mean age (years)	50	55	59
SD	16	16	13

**Table 2:** Chronic pancreatitis etiologies for each group of patients.

	Group A (Without PEI)	Group B (With Mild to Moderate PEI)	Group C (With Severe PEI)
CFTR mutation	5	8	-
SPINK-1 mutation	1	1	-
Obstructive Pancreatitis	10	14	7
Autoimmune Pancreatitis	8	11	28
Paraduodenal Pancreatitis	2	-	3
Pancreas Divisum	4	-	2
Idiopathic	11	6	2
Hypertriglyceridaemia	1	-	-

**Table 3:** Receiver-operator curve (ROC) analysis for diagnosing PEI (pancreatic exocrine insufficiency) using PMS analysis.

Comparison	AUROC	*p*-Value	Threshold (PMS)	Sensitivity (%)	Specificity (%)
Group A vs. B-C (pts without vs. pts with mild or severe PEI)	0.8373	<0.0001	1.4	95.12	56.10
Group A-B vs. C (pts without or with mild vs. pts with severe PEI)	0.8094	<0.0001	1.005	97.62	55.56
Groups A vs. B (pts without vs. pts with mild PEI)	0.7701	<0.0001	1.205	80	64.98

## Data Availability

The data presented in this study are available on request from the corresponding author. The data are not publicly available due to privacy.
